# Dry eye: A hospital-based sociodemographic, risk and clinical classification profile

**DOI:** 10.4102/hsag.v30i0.2793

**Published:** 2025-02-21

**Authors:** Phindile P. Mdlalose, Vanessa R. Moodley

**Affiliations:** 1 Department of Optometry, Faculty of Health Sciences, University of KwaZulu-Natal, Durban, South Africa; 2 Keratoconus Foundation South Africa, Durban, South Africa

**Keywords:** dry eye, prevalence, SPEED Questionnaire, Schirmer 2, TBUT

## Abstract

**Background:**

Dry eye, a multifactorial disease of the tears and ocular surface, is considered a significantly growing public health problem worldwide.

**Aim:**

This study aimed to determine the prevalence and risk factors of dry eye disease (DED) in a population attending an eye hospital in KwaZulu-Natal, South Africa.

**Setting:**

The study was conducted at McCord Provincial Eye Hospital in Durban, KwaZulu-Natal, South Africa.

**Methods:**

A cross-sectional hospital-based study was conducted between July 2023 and August 2023. Diagnosis was confirmed with a SPEED score of ≥ 4 and a monocular tear break up time (TBUT) ≤ 10 s or Schirmer 2 ≤ 15 mm/5 min. Data were managed using Statistical Package for Social Sciences (SPSS) software version 28.

**Results:**

The overall prevalence of DED was 82.3% and highest in those > 65 years of age (odds ratios [OR] = 3.17; 95% confidence intervals [CI]: 1.45–6.94; *p* = 0.04). Risk factors significantly associated with DED were age (*p* < 0.009), systemic diseases (diabetes [*p* < 0.01], HIV [*p* < 0.02], hypertension [*p* < 0.02] and medication [*p* < 0.01]).

**Conclusion:**

The study revealed a high DED prevalence, which increased with age. Diabetes, hypertension and HIV were identified as significant risk factors for DED. Routine tear function evaluation should be an integral part of the assessment protocols of these highly susceptible patients. Furthermore, public health education in DED is essential to help reduce prevalence through the promotion of behaviour modifications.

**Contribution:**

This study provides knowledge regarding the prevalence and risk factors of DED in KwaZulu-Natal, South Africa.

## Introduction

Dry eye, a multi-factorial disease of the tears and ocular surface, is one of the most common reasons that patients visit eye care professionals (Bradley et al. 2019:225). The disease has been defined in a variety of ways such as dry eye disease (DED), keratoconjunctivitis sicca (KCS), dry eye syndrome (DES) or dysfunctional tear syndrome (Huang et al. 2022:3254).

The tear film made up of the lipid, aqueous and mucin layers, is very important in lubricating, nourishing and protecting the ocular surface (Yazdani et al. 2023:3534). If any of these layers are lacking or deficient in composition and remain untreated, a person will experience dry eye symptoms and display related signs. Common symptoms include sandy sensation, itching, burning, increased corneal sensitivity, tearing, intermittent blurry vision and reduced visual performance (Lemp 2008:350–351; Onua & Chukwuka 2017:96).

Factors causing dry eye include medication, autoimmune diseases (lupus, arthritis and Sjogren’s syndrome), ageing, and hormonal and environmental factors (Matossian et al. 2019:503). Contributing pharmaceuticals include antihistamines, antidepressants, oral contraceptives, Beta-blockers and diuretics. Contact lens wear, visual display units (VDUs), refractive surgery, smoking and diet have also been identified as factors linked to DED (Ang, Dartt & Tsubota 2001:318–322; Ezinne et al. 2023:37–38).

Dry eye, if not treated can have a measurable impact on various aspects of a person’s quality of life (QoL), including workplace productivity, psychological functioning, physical, socioeconomic, social and daily activities (Matossian et al. 2019:502). Depression, stress and anxiety have also been correlated with impaired vision-related QoL in dry eye patients (Yilmaz et al. 2015:626; Zhou et al. 2022:392).

As a highly prevalent ocular surface disease, dry eye is considered a growing public health problem worldwide. Surveys around the world estimate DED prevalence as ranging from 9.5% to 90% in various age groups across different countries (Lemp 2008; Shanti et al. 2020:1), with population-based studies ranging from 5.7% to 57.5% (Onua & Chukwuka 2017:99) and in hospital-based studies from 17.9% to 29.9% (Onwubiko et al. 2014:157). Large variations may be because of variations in population demographic profiles and geographic locations with different environmental exposures and a variation of diagnostic criteria as used (Shanti et al. 2020:1).

Recent studies reveal a significant DED burden in Africa, estimating a 42% prevalence (Akowuah and Kobia-Acquah 2020:1096; Osae et al. 2017:165). In addition, Onua and Chukwuka (2017:95) assert that even though DED is a known common eye disease worldwide, many people, especially in underdeveloped countries, remain undiagnosed and untreated. In South Africa, because of the unaffordability of private healthcare, 73% of households utilise the under-resourced, overly burdened, public sector, for their healthcare services (Cowling 2023; Rensburg 2021). The sector is plagued by a shortage of medical professionals, a high number of health professionals emigrating, inequality and elevated rates of foreign migration into the country (Rensburg 2021). Dry eye prevalence in South Africa is not well-represented in the scholarly research, with only one hospital-based study done in Eastern Cape province (Nonkula, 2019). No information on DED prevalence or management within the public health sector in KwaZulu-Natal (KZN) is currently reported. As McCord Provincial Eye Hospital (MPEH) is a tertiary hospital and the referral centre for eye diseases for eleven districts in KwaZulu-Natal, this study aimed to determine the prevalence, severity, classification and risk factors of DED in a hospital-based population. Additionally, DED will be classified according to the Tear Film and Ocular Surface Society Dry Eye Workshop (TFOS DEWS) which classifies dry eye into three categories: aqueous deficient dry eye (ADDE), evaporative dry eye (EDE) and mixed, when there is an overlap between the two types (Craig et al. 2017:281).

## Research methods and design

### Study design

A quantitative, cross-sectional, descriptive study was conducted among patients attending MPEH, KZN, South Africa. For efficient data collection and a high responsive rate, a hospital-based setting was chosen because of the existing infrastructure and accessible population.

### Study population and sampling

For this study, a minimum sample size of 600 patients was determined using the formula: *n = Z*^2^ *p(1-q) (DEFF)/d*^2^.

Six hundred and two consenting participants using convenient sampling were enrolled in the study in a 2-month period (July 2023–August 2023). These patients were recruited for the study as they were waiting to be seen by the doctor at MPEH. Males and females, seven years and older, presenting at the hospital, were included. Patients with any ocular surface pathology or any history of intellectual impairment were excluded. Dry eye disease presence was investigated through self-reported DED symptoms and evaluation of clinical signs. The TFOS DEWS II-recommended diagnostic criteria were applied to diagnose DED (Wolffsohn et al. 2017:539–574).

### Data collection

Demographic data (age, gender, occupation, medical and ocular history) was recorded for each participant by the researcher. The validated Standardised Patient Evaluation of Eye Dryness (SPEED) Questionnaire was administered by a researcher, with participants rating their ocular symptoms, frequency and severity.

Each patient initially underwent a slit lamp evaluation to assess the overall ocular surface before the commencement of dry eye testing. Tear break up time (TBUT) and Schirmer 2 tests were then performed. A TBUT score of < 10 s was indicative of tear film instability DED. The Schirmer 2 test measurements were recorded after 5 min with less than 15 mm/5 min considered diagnostic of DED. Tests were performed and recorded independently for both eyes by a researcher.

Dry eye disease diagnosis was confirmed by the presence of subjective DED symptoms, revealed by a SPEED score of ≥ 4, combined with at least one of the objective DED signs in the worse eye that is TBUT ≤ 10 sec or Schirmer 2 ≤ 15 mm/5 min. Patients diagnosed with DED were further categorised into ADDE, EDE and mixed, with EDE participants having abnormal TBUT and normal Schirmer findings. Those with normal TBUT and abnormal Schirmer were classified as ADDE, while those classified as mixed had both TBUT and Schirmer being abnormal. Dry eye disease was further categorised by severity grades, which are mild, moderate and severe. Participants who required treatment for DED were referred for management and further review.

### Data analysis

Data from questionnaires and clinical evaluation were captured and analysed by a statistician within a quantitative framework, using Statistical Package for Social Sciences (SPSS) version 28.0 software. Univariate analysis checked central tendency (mean, median and mode), range, maximum and minimum values, and standard deviation. Relative risks were estimated as odds ratios (OR) at 95% confidence intervals (CI). A *p*-value of *< 0.05* was considered statistically significant. A Pearson Chi-square and Fischer exact test of association was used for any association between categorical variables.

### Ethical considerations

Ethical approval was obtained from the Biomedical Research Ethics Committee (BREC) at the University of KwaZulu-Natal, with consent received on 21 June 2023. The ethics approval number is BREC/00003439/2021. Permission to conduct the study was also obtained from the Department of Health, KwaZulu-Natal province (KZ-20211-033). A letter of support was received from the eThekwini district, and a gatekeeper letter to conduct research at MPEH was provided by the CEO. The study adhered to the tenets of the Helsinki Declaration. Participants received a full explanation of the procedures, and their confidentiality was ensured. All individual participants involved in the study provided written consent to participate, and participation was voluntary.

## Results

Six hundred and two patients participated in this study with ages ranging from 7 to 88 years (mean = 48.54 years +18.76) (Table 1). Females (*n* = 473; 78.6%) and participants of African ethnicity (82.0%; *n* = 501) were in the majority, with the remaining ethnic groups (Whites, Indians and Mixed-Race) combined, only comprising 18% (*n* = 101).

**TABLE 1:** Sociodemographic characteristics of participants.

Variables	*n*	%
**Age (years)**		
7–18	62	10.2
19–40	159	26.4
41–55	158	26.4
56–65	103	17.1
>65	120	19.9
**Gender**		
Female	473	78.6
Male	129	21.4
**Occupation**		
Employed	118	19.6
Scholar	76	12.6
Pensioners	187	31.1
Unemployed	221	36.7
**Systemic diseases**		
Yes	333	55.3
No	269	44.7
**Medication (systemic and ocular)**		
Yes	327	54.3
No	275	45.7
**Systemic diseases**		
Hypertension	176	29.2
Diabetes	99	16.4
HIV	78	13
Glaucoma	46	7.6
Vernal keratoconjunctivitis	13	2.2
Arthritis	14	2.3
Other	75	12.5
None	101	16.7
**Other**		
Sleep Apnoea	102	17
Contact lens	6	1
Previous surgery	107	17.8

*n*, number; %, percentage; VKC, vernal keratoconjunctivitis; HIV, human immunodeficiency virus.

The overall prevalence of DED was 83.2% (*n* = 501), affecting 398 (79.4%) females and 103 (20.6%) males. Dry eye disease significantly increased with age (*p = 0.009*), with the majority affected being in the 19–40 and 41–55 years age range (Table 2).

**TABLE 2:** Prevalence of dry eye disease according to sociodemographic characteristics of participants.

Variable	Dry eye (No)	%	Dry eye (Yes)	%	Total	*p*
**Age (years)**						
7–18	19	30.6	43	69.4	62	0.02*
19–40	39	24.5	120	75.5	159	-
41–55	18	11.4	140	88.6	158	-
56–65	11	10.7	92	89.3	103	-
>65	14	11.7	106	88.3	120	-
**Gender**						
Female	75	15.9	398	84.1	473	0.25
Male	26	20.2	103	79.8	129	-
**Occupation**						
Employed	21	17.8	97	82.2	118	0.03*
Scholar	20	26.3	56	73.7	76	-
Pensioner	21	11.2	166	88.8	187	-
Unemployed	39	17.6	182	82.4	221	-
**Indoor or outdoor**						
Indoors	37	21.1	138	78.9	175	0.13
Outdoors	3	23.1	10	76.9	13	-
N/A	61	14.7	353	85.3	414	-
**Race**						
A	90	18.0	411	82.0	501	0.08
M-R/I/W	11	10.9	90	89.1	101	-
**Systemic diseases**						
No	57	21.3	211	78.7	268	0.006**
Yes	43	12.9	290	87.1	333	-
**Comorbidities**						
Diabetes						
No	93	18.5	410	81.5	503	0.01**
Yes	8	8.1	91	91.9	99	-
**Glaucoma**						
No	96	17.3	460	82.7	556	0.26
Yes	5	10.9	41	89.1	46	-
**Hypertension**						
No	81	19.0	345	81.0	426	0.02*
Yes	20	11.4	156	88.6	176	-
**Arthritis**						
No	101	17.1	488	82.9	589	0.1
Yes	0	0.0	13	100.0	13	-
**HIV**						
No	95	18.1	429	81.9	524	0.02*
Yes	6	7.7	72	92.3	78	-
**Previous eye surgery**						
No	82	16.6	413	83.4	495	0.76
Yes	19	17.8	88	82.2	107	-
**Medication**						
No	58	21.1	217	78.9	275	0.009**
Yes	43	13.1	284	86.9	327	-
**Sleep apnoea**						
No	86	17.2	414	82.8	500	0.54
Yes	15	14.7	87	85.3	102	
**Contact lens**						
No	100	16.8	496	83.2	596	0.9
Yes	1	16.7	5	83.3	6	-

Note: Significance level (*p*-value):

* , *p* < 0.05;

** , *p* < 0.001.

DED, dry eye disease; %, percentage; A, African; I, Indian; M-R, mixed race; W, white; HIV, human immunodeficiency virus.

The overall prevalence of DED determined in the study was 83.2%. This applied to patients having failed the SPEED assessment and at least one of the clinical tests.

Analysis of the TBUT and Schirmer 2 tests was undertaken to classify DED according to type and severity as shown in Table 3a and Table 3b.

**TABLE 3a:** Dry eye disease according to type and severity.

Type of DED					
ADDE		EDE		Mixed	
*n*	%	*n*	%	*n*	%
16	3.2	314	62.7	171	34.1

DED, dry eye disease; ADDE, aqueous deficient dry eye; EDE, evaporative dry eye.

**TABLE 3b:** Dry eye disease according to type and severity.

Severity of DED					
Mild		Moderate		Severe	
*n*	%	*n*	%	*n*	%
119	23.8	227	45.3	155	30.9

DED, dry eye disease.

Similar to the overall prevalence trend, the TBUT test revealed that the majority of participants had moderate (*n* = 112) or severe (*n* = 328) DED as compared to those with mild DED or normal findings *(p < 0.001)*. The prevalence of dry eye according to the Schirmer 2 test was 52.8% (*n* = 318) and as with the TBUT and SPEED score severity analysis, there was a statistically significant difference *(p < 0.001)* in DED severity, with the majority having mild (*n* = 132) or moderate (*n* = 124) DED.

The SPEED Questionnaire, a valid and reliable instrument for measuring dry eye severity revealed a mean SPEED value of 6.05 (s.d. = 4.39), which is greater than the defined cut-off points for dry eye diagnosis (> 4). Of the 602 participants, the majority (*n* = 337; 56%) had DED with > 4 SPEED scores (Table 4).

**TABLE 4:** Severity of dry eye disease according to the standardised patient evaluation of eye dryness, tear break up time and Schirmer 2 tests.

SPEED score	*n*	%	*p*	TBUT severity	*n*	%	*p*	Schirmer 2 severity	*n*	%	*p*
Normal or mild (0–4)	209	41.7	< 0.001*	Mild	45	8.9	< 0.001*	Mild	132	26.3	< 0.001*
Moderate (5–7)	137	27.3	-	Moderate	112	23.4	-	Moderate	124	24.8	-
Severe (8–28)	155	31.0	-	Severe	328	65.5	-	Severe	26	5.2	-

Note: Significance level (*p*-value):

* , *p* < 0.001.

SPEED, standardised patient evaluation of eye dryness; TBUT, tear break up time.

As shown in Table 5, systemic diseases (*p = 0.006*) and medication (*p = 0.009*) were factors with highly significant associations with DED.

**TABLE 5:** Risk factors significantly associated with dry eye disease.

Variables	*n*	*p*	OR	95%CI
**Age (years)**				
19–65	350	0.02*	2.12	1.16–3.90
> 65	105	0.04*	3.17	1.45–6.94
**Occupation**				
No	101	-	-	-
Yes	501	0.03*	1.17	0.89–3.29
**Medication**				
No	217	-	-	-
Yes	284	0.009**	1.77	1.15–2.72
**Systemic disease**				
No	211	-	-	-
Yes	290	0.006**	1.82	1.18–2.81
**Hypertension**				
No	345	-	-	-
Yes	156	0.02*	1.83	1.08–3.09
**Diabetes**				
No	410	-	-	-
Yes	91	0.01**	2.58	1.21–5.50
**HIV**				
No	429	-	-	-
Yes	72	0.03*	2.66	1.12–6.29

Note: Significance level (*p*-value):

* , *p* < 0.05;

** , *p* < 0.01.

CI, confidence intervals; OR, odds ratios; *n*, number; OR, odds ratio.

## Discussion

Few studies have investigated dry eye prevalence in South Africa. This is the first hospital-based study regarding DED’s prevalence and risk factors in KZN. This study focussed on determining the prevalence and risk factors of DED in a hospital-based population in KZN, South Africa. The study combined subjective (dry eye symptom questionnaire) and objective clinical tests to confirm the diagnosis of DED based on the TFOS DEWS II diagnostic criteria (Wolffsohn et al. 2017:539–574) as mentioned earlier. Sociodemographics, associated risks and clinical profiles with the development of DED were also evaluated.

The prevalence of DED, in this hospital-based study was 83.2%, similar to the 92% (Ocular surface disease index – OSDI) and 63% (TBUT and Schirmer 2) found in another South African study (Nonkula 2019). Shah and Jani (2015:152) in a study including participants aged ≥ 40 years, also revealed a prevalence of 67.3% in those ≥ 71 years. However, two other South African studies (Castelyn et al. 2016:6; Gillan 2009:190), found lower prevalence ranging from 40% to 60%. The difference could be because of the wider age range of this study’s participants, which was 7 to 88, as compared to their younger, healthy, university-based study population.

This study site is based in an urban environment and although noting that lifestyle, diet and air pollution may contribute to the high DED prevalence (Wang et al. 2021:101409), the impact of these specific variables was not investigated in this study. Further investigations are warranted as other studies conducted in different parts of the country also had similar prevalence findings.

The symptom SPEED questionnaire is often used to detect the condition early; however, Shimazaki (2018:11) asserts that there is no correlation between a patient’s ocular symptoms, as reported on dry eye questionnaires, and signs detected through clinical tests. The assertion is supported by findings in this study, which revealed a DED prevalence of 56%, based on the SPEED symptom questionnaire and 83.2% according to clinical tests. Some participants with DED clinical signs did not report symptoms or had few symptoms, implying that they possibly consider the symptoms as being normal experiences. Dry eye disease prevalence in asymptomatic participants was 41.7%, which may be because of the natural inconsistency of the disease process, the nature of symptoms and differences in pain thresholds (DEWS 2007:75; Stapleton et al. 2017:341). Although it may be an effective population-based screening tool, the SPEED Questionnaire, as an in-practice clinical screening tool, may miss asymptomatic DED participants. It is advised that, in addition to the SPEED Questionnaire, a clinical test such as TBUT or Schirmer should be performed on all hospital outpatients to rule out DED. Additionally, SPEED or any other dry eye symptom questionnaire should be administered at all levels (primary, secondary and tertiary) of healthcare for dry eye screening.

This study, as in other studies (Shah & Jani 2015:154), found DED to be significantly associated with age (*p = 0.02*), with multivariate analysis finding that adults > 65 years are three times more likely to be diagnosed with DED. Numerous other studies also showed that adults of > 60 years of age are more likely to be diagnosed with DED than younger individuals (Farrand et al. 2017:94; Martinez et al. 2016:1337). These findings could be because of decreased tear secretion, increasing meibomian gland dysfunction and thinning of the lipid layer with advancing age (Bhatt et al. 2023:1456; Li et al. 2023:214). However, some studies (Betiku et al. 2022:359; Bhatt et al. 2023:1456; Schaumberg et al. 2003:318) reported an increase in the prevalence of DED among adults aged <50 years, with researchers ascribing the cause to high levels of mobile devices and VDU’s usage in this younger population. It is recommended that digital device usage be included in future DED studies among this population demographic.

The study found a higher prevalence in females (79.3%) than in males (20.6%), although not statistically significant (*p = 0.25*), which has also been found in other studies (Farrand et al. 2017:94; Schaumberg et al. 2003:318; Sharma & Hindman 2014:104). Clayton and Davis (2015:103) noted that DED is generally present in women approximately six years earlier than in men. According to Versura, Giannaccare and Campos (2015:166), DED is most likely to be related to an imbalance of SSHs (sex steroid hormones) associated with advanced age, mainly menopause. Additionally, hormonal influences are reported to play an important role in the development of DED among females as female sex hormones affect both lacrimal and meibomian glands (Bhatt et al. 2023:1456). Therefore, targeted education is recommended to ensure that females are timeously advised and treated.

The study explored and found a significant association between those in employment and DED (*p = 0.03*). Findings may be because of increased air conditioner exposure at workplaces, exposure to VDU device screen times of ± 8 h and reduced blink rate. Modifying environmental conditions, using tear supplements, intermittently looking away from the device screen and doing blinking exercises will aid in managing the condition.

This study had 36.7% (*n* = 221) unemployed participants, of which 182 had DED. In a challenging economic context, unemployment is a personal situation which may lead to DED caused by mental health issues such as stress and anxiety. Several studies support this notion (Liu et al. 2022:930714; Stapleton et al. 2017:355; Yilmaz, Gokler & Unsal 2015:626), which reported that people with depression, anxiety or stress are more likely to have DED. In taking a holistic approach to the health and well-being of their patients, clinicians should obtain the employment history and include DED investigations where appropriate.

A significant number (348) of pensioners and unemployed individuals, dependent on the public sector for healthcare services had DED. Noting that tear supplements may be unaffordable for these individuals, patients should be educated on behaviour modification strategies, and public health facilities should dispense dry eye medication where clinically indicated. It has been noted that medication for dry eye is limited even at the tertiary facility (MPEH) which all facilities refer to for severe dry eye cases. Practitioners in primary care and hospitals are limited as not much is available for dry eye as the facilities prescribe according to what is listed in the essential medicines list (EML). Essential medicine list with supporting Standard Treatment Guidelines (STGs) has been developed for different levels of care by different committees from National Department of Health (STG & EML 2019:24). A lack of dry eye medication may potentially have a negative impact on the individual QoL, affecting the social and psychological functioning and daily living activities (Liu et al. 2022:930714; Pouyeh et al. 2012:1061; Stapleton et al. 2017:355). As the long-term costs for DED management may cause an increased financial burden on the state and on individuals (Stapleton et al. 2017:354; Uchino & Schaumberg 2013:55; Yang et al. 2021:771352; Yu, Asche & Fairchild 2011:379), preventative strategies could also be developed by relevant stakeholders and incorporated into public eye health awareness campaigns.

Dry eye disease, most common in older participants, was significantly associated *(p = 0.006)* with systemic diseases such as diabetes, glaucoma, hypertension and HIV, as also found in other studies (Kam et al. 2023:1263; Kawashima 2018:138; Zhang et al. 2016:8201053). The DED association was also found in the hospital-based study conducted in Mthatha, Eastern Cape province (Nonkula 2019), which included a large number (86%) of older people (>40 years) presenting with a history of systemic diseases and being on chronic medication.

Diabetes is one of the leading systemic risk factors for DED (Zhang et al. 2016:8201053), a finding confirmed in this study. The study had 99 (16.4%) participants with diabetes, of whom 91 (92%) had DED and were almost three times more likely to have DED as compared to non-diabetics (OR = 2.58; 95% CI: 121–550). Dry eye disease could be caused by tear film dysfunction, instability of the tear film, secretion deficiency, decrease in the lipid layer thickness and corneal sensitivity in people with diabetes (Bhatt et al. 2023:1457; Zhang et al. 2016:8201053). Noting the increasing prevalence of diabetes globally (Sidahmed et al. 2023:324; Standl et al. 2019:8), it is advised that, in clinical practice, dry eye screening and tear function evaluations be part of the ophthalmic assessment routine for patients with diabetes.

Hypertension is also one of the systemic diseases known to be associated with DED (Kamil 2020:129) and was found to be significantly associated (*p = 0.02*) in this study. According to Kalkan Akcay et al. (2015:117), Angiotensin Converting Enzyme (ACE) inhibitors and Beta-blockers used in treating hypertension can cause DED. Therefore, it is advised that clinicians consider the risk of DED when prescribing such medication (Kim et al. 2015:241), assess for dry eye and where indicated prescribe tear supplements. Similarly, when clinicians consult patients on anti-hypertension medication, they should ask the patient about DED and make a note to evaluate for DED presence during ocular examination.

Dryness of the ocular surface has a reported prevalence of 11%–50% among individuals with HIV and AIDS and has been attributed to vitamin A deficiency and long-term use of antiretroviral therapy (ART) (Gichuhi & Arunga 2020:76). This study also revealed a significant association with HIV (*p = 0.03*) with 72 (92%) of the HIV-positive patients having DED, highlighting that the ability to produce tears is compromised in HIV-positive patients. Dry eye disease screening for all HIV patients who visit the hospital should be part of their routine investigative clinical assessment.

Glaucoma was also significantly associated with DED, as 89.1% of affected patients had DED. According to Kam et al. (2023:12687), ocular surface glaucoma diseases are mostly related to inflammatory reactions to preservatives and, occasionally, allergic reactions to some medications. Preservative-free artificial tears and/or non-preserved anti-glaucoma drugs are recommended to improve the hyperosmolarity of the tear film (Shah & Jani 2015:154). Practitioners should normalise screening patients presenting with these diseases for DED and managing appropriately with medication that does not exacerbate DED.

According to the American Academy of Ophthalmology (2013), a collection of systemic medications is known to exacerbate DED symptoms (such as burning sensation, tearing or photophobia). The study revealed a significant association between systemic medication and DED prevalence, findings similar to that of Betiku et al. (2022:359). A thorough case history should be taken in patients with systemic diseases to obtain information about medication and their respective side effects. This will aid in educating patients where medication may be the contributing factor to DED development. Alternative medication could be recommended through their respective primary healthcare providers.

The majority of participants had moderate to severe DED, which is of concern as it is known to negatively impact the individual’s QoL (Matossian et al. 2019:502; Pouyeh et al. 2012:106). Noting the severity, this warrants a need for MPEH to provide treatment such as artificial tears, offer patient education (modifying their environment, lifestyle and diet) and home remedies (such as lid scrubs and warm compressors) and investigate and implement preventative systemic disease-related interventions.

Although this study found no significant association between DED and cataract surgery *(p = 0.76)*, Ishrat, Nema and Chandravanshi (2019:39) highlight that ocular surgeries (refractive or cataract) may induce or exacerbate DED. Pre-op DED assessment is therefore important to ensure accurate measurements for surgical planning. Further, post-op DED management is essential as, if not treated, it may delay wound healing (Naderi, Gormley & O’Brart 2020:850).

### Limitations

The study was a single-centre, hospital-based study which could increase the reported prevalence of a disease condition compared to a population-based prevalence study. The small sample size and/or the 2-month study period may limit generalisability.

## Conclusion

The prevalence of DED in the largest eye hospital in KwaZulu-Natal was found to be high. Dry eye disease prevalence increased with age, and diabetes, hypertension, HIV and glaucoma were the identified systemic disease risk factors. Routine tear function investigations should be an integral part of the health assessments of these high-risk patients. Furthermore, public health education, which includes information on DED symptoms and signs, risk factors and treatment options, including home remedies (lid hygiene, warm compressors, lifestyle and diet) and behaviour modification strategies should be considered as part of eye health promotion programmes. The symptom SPEED questionnaire should ideally be combined with tear function clinical screening tests to identify patients with DED. As the study was conducted in a coastal facility, further studies should be carried out in inland hospitals to provide additional data on environmental variations. Furthermore, noting the high prevalence (83.2%) of DED found in this facility-based study, MPEH which is a provincial eye hospital in KZN, supported by the Department of Health, should consider having a special dry eye unit, towards the provision of a more comprehensive eye health service to patients. Dry eye disease is so prevalent that it should be cared for in all services in the hospital as well as in primary care and general hospital care. Furthermore, this emphasises the importance of healthcare professionals administering dry eye symptom questionnaires at all levels of healthcare which are primary (primary healthcare and community health centre), secondary and tertiary. This is particularly crucial at the primary level, where professionals can refer patients for further investigation and management as needed. Data from this study can used to develop clinical guidelines for DED management for practitioners in primary care and general hospital practice by engaging with the National Department of Health guideline structures and committees.

## References

[CIT0001] Akowuah, P.K. & Kobia-Acquah, E., 2020, ‘Prevalence of dry eye disease in Africa: A systematic review and meta-analysis’, *Optometry and Vision Science* 97(12), 1089–1098. 10.1097/OPX.0000000000001610 33259380

[CIT0002] American Academy of Ophthalmology, 2013, ‘Dry eye syndrome preferred practice’, in *American Academy Ophthalmology Annual Meeting*, 16–19 November 2013, viewed 21 September 2013, from https://www.aao.org/preferred-practice-pattern/dry-eye-syndrome-ppp—2013.

[CIT0003] Ang, R.T., Dartt, D.A. & Tsubota, K., 2001, ‘Dry eye after refractive surgery’, *Current Opinion in Ophthalmology* 12(4), 318–322. 10.1097/00055735-200108000-00013 11507347

[CIT0004] Betiku, A.O., Oduyoye, O.O., Jagun, O.O., Olajide, O.S., Adebusoye, S.O. & Aham-Onyebuchi, U.O., 2022, ‘Prevalence and risk factors associated with dry eye disease among adults in a population-based setting in South-West Nigeria’, *Nigerian Journal of Clinical Practice* 25(3), 354–360. 10.4103/njcp.njcp_1598_21 35295060

[CIT0005] Bhatt, K., Singh, S., Singh, K., Kumar, S. & Dwivedi, K., 2023, ‘Prevalence of dry eye, its categorization (Dry Eye Workshop II), and pathological correlation: A tertiary care study’, *Indian Journal of Ophthalmology* 71(4), 1454–1458. 10.4103/IJO.IJO_2591_22 37026281 PMC10276703

[CIT0006] Bradley, J.L., Stillman, I.Ö., Pivneva, I., Guerin, A., Evans, A.M. & Dana, R., 2019, ‘Dry eye disease ranking among common reasons for seeking eye care in a large US claims database’, *Clinical Ophthalmology* 13, 225–232. 10.2147/OPTH.S188314 30774303 PMC6362914

[CIT0007] Castelyn, B., Majola, S., Motilal, R., Naidu, M.T., Ndebele, S.A., Vally, T.A. et al., 2015, ‘Prevalence of dry eye amongst black and Indian university students aged 18–30 years’, *African Vision and Eye Health* 74(1), 6. 10.4102/aveh.v74i1.14

[CIT0008] Clayton, J.A. & Davis, A.F., 2015, ‘Sex/gender disparities and women’s eye health’, *Current Eye Research* 40(2), 102–109. 10.3109/02713683.2014.986333 25548854

[CIT0009] Cowling, N., 2023, *Distribution of health care choices of households in South Africa*, viewed 20 September 2023, from www.statista.com.

[CIT0010] Craig, J.P., Nichols, K.K., Akpek, E.K., Caffery, B., Dua, H.S., Joo, C.K. et al. 2017, ‘TFOS DEWS II definition and classification report’, *The Ocular Surface* 15(3), 276–283. 10.1016/j.jtos.2017.05.008 28736335

[CIT0011] Ezinne, N., Alemu, H.W., Cheklie, T., Ekemiri, K., Mohammed, R. & James, S., 2023, ‘High prevalence of symptomatic dry eye disease among university students during the COVID-19 pandemic in University of West Indies, Trinidad and Tobago’, *Clinical Optometry* 2023, 37–43. 10.2147/OPTO.S396135 PMC9990450 36896339

[CIT0012] Farrand, K.F., Fridman, M., Stillman, I.Ö. & Schaumberg, D.A., 2017, ‘Prevalence of diagnosed dry eye disease in the United States among adults aged 18 years and older’, *American Journal of Ophthalmology* 182, 90–98. 10.1016/j.ajo.2017.06.033 28705660

[CIT0013] Gichuhi, S. & Arunga, S., 2020, ‘Viral diseases of the eye’, *Journal of Community Health Research* 33(108), 76–78. PMC7205170 32395031

[CIT0014] Gillan, W.D.H., 2009, ‘A small-sample survey of dry eye symptoms using the Ocular Surface Disease Index’, *African Vision and Eye Health* 68(4), 188–191. 10.4102/aveh.v68i4.167

[CIT0015] Huang, R., Su, C., Fang, L., Lu, J., Chen, J. & Ding, Y., 2022, ‘Dry eye syndrome: Comprehensive etiologies and recent clinical trials’, *International Ophthalmology* 42(10), 3253–3272. 10.1007/s10792-022-02320-7 35678897 PMC9178318

[CIT0016] Ishrat, S., Nema, N. & Chandravanshi, S.C.L., 2019, ‘Incidence and pattern of dry eye after cataract surgery’, *Saudi Journal of Ophthalmology* 33(1), 34–40. 10.1016/j.sjopt.2018.10.009 30930661 PMC6424692

[CIT0017] Kalkan Akcay, E., Akcay, M., Can, G.D., Aslan, N., Uysal, B.S., Ceran, B.B. et al., 2015, ‘The effect of antihypertensive therapy on dry eye disease’, *Cutaneous and Ocular Toxicology* 34(2), 117–123. 10.3109/15569527.2014.912660 24938452

[CIT0018] Kam, K.W., Di Zazzo, A., De Gregorio, C., Narang, P., Jhanji, V. & Basu, S., 2023, ‘A review on drug-induced dry eye disease’, *Indian Journal of Ophthalmology* 71(4), 1263–1269. 10.4103/IJO.IJO_2782_22 37026257 PMC10276716

[CIT0019] Kamil, Z., 2020, ‘Hypertension and dry eye’, *Journal of Surgery Pakistan* 25(3), 127–130.

[CIT0020] Kawashima, M., 2018, ‘Systemic health and dry eye’, *Investigative Ophthalmology & Visual Science* 59(14), DES138–DES142. 10.1167/iovs.17-23765 30481818

[CIT0021] Kim, Y.S., Sun, H.J., Kim, T.H., Kang, K.D. & Lee, S.J., 2015, ‘Ocular manifestations of acquired immunodeficiency syndrome’, *Korean Journal of Ophthalmology* 29(4), 241–248. 10.3341/kjo.2015.29.4.241 26240508 PMC4520867

[CIT0022] Lemp, M.A. & Foulks, G.N., 2007, ‘The definition and classification of dry eye disease’, *Ocular Surface* 5(2), 75–92. 17508116 10.1016/s1542-0124(12)70081-2

[CIT0023] Lemp, M.A., 2008, ‘Advances in understanding and managing dry eye disease’, *American Journal of Ophthalmology* 146(3), 350–356. 10.1016/j.ajo.2008.05.016 18599017

[CIT0024] Li, X., Wang, Z., Mu, J., Puerkaiti, H., Nulahou, A., Zhang, J. et al., 2023, ‘Prevalence and associated risk factors of dry eye disease in Hotan, Xinjiang: A cross-sectional study’, *BMC Ophthalmology* 23(1), 214. 10.1186/s12886-023-02955-9*96-37189099 PMC10184355

[CIT0025] Liu, Z., Sun, S., Sun, X., Wu, Y. & Huang, Y., 2022, ‘Differences of anxiety and depression in dry eye disease patients according to age groups’, *Frontiers in Psychiatry* 13, 930714. 10.3389/fpsyt.2022.930714 35911246 PMC9326042

[CIT0026] Martinez, J.D., Galor, A., Ramos-Betancourt, N., Lisker-Cervantes, A., Beltrán, F., Ozorno-Zárate, J. et al., 2016, ‘Frequency and risk factors associated with dry eye in patients attending a tertiary care ophthalmology center in Mexico City’ *Clinical Ophthalmology* 10, 1335–1342. 27499613 10.2147/OPTH.S106451 PMC4959589

[CIT0027] Matossian, C., McDonald, M., Donaldson, K.E., Nichols, K.K., MacIver, S. & Gupta, P.K., 2019, ‘Dry eye disease: Consideration for women’s health’, *Journal of Women’s Health* 28(4), 502–514. 10.1089/jwh.2018.7041 PMC6482917 30694724

[CIT0028] Naderi, K., Gormley, J. & O’Brart, D., 2020, ‘Cataract surgery and dry eye disease: A review’, *European Journal of Ophthalmology* 30(5), 840–855. 10.1177/1120672120929958 32515220 PMC7549290

[CIT0029] National Department of Health, South Africa, 2019, *Essential drugs programme hospital level (adults) standard treatment guidelines and essential medicines list*, 5th edn., pp. 1–694, National Department of Health, viewed 18 January 2020, from http://www.health.gov.za/edp.php.

[CIT0030] Nonkula, D.M., 2019, ‘The prevalence of dry eye syndrome among patients at the eye clinic in Nelson Mandela Academic Hospital’, unpublished dissertation, University of the Free State, viewed 30 January 2019, from https://ufs.ac.za.

[CIT0031] Onwubiko, S.N., Eze, B.I., Udeh, N.N., Arinze, O.C., Onwasigwe, E.N. & Umeh, R.E., 2014, ‘Dry eye disease: Prevalence, distribution and determinants in a hospital-based population’, *Contact Lens and Anterior Eye* 37(3), 157–161. 10.1016/j.clae.2013.09.009 24126152

[CIT0032] Onua, A.A. & Chukwuka, I.O., 2017, ‘Prevalence of dry eye disease in a rural Niger delta community, southern Nigeria’, *Open Journal of Ophthalmology* 7(02), 95–102. 10.4236/ojoph.2017.72014

[CIT0033] Osae, A.E., Gehlsen, U., Horstmann, J., Siebelmann, S., Stern, M.E., Kumah, D.B. et al., 2017, ‘Epidemiology of dry eye disease in Africa: The sparse information, gaps and opportunities’, *The Ocular Surface* 15(2), 159–168. 10.1016/j.jtos.2017.01.001 28065724

[CIT0034] Pouyeh, B., Viteri, E., Feuer, W., Lee, D.J., Florez, H., Fabian, J.A. et al., 2012, ‘Impact of ocular surface symptoms on quality of life in a United States Veterans affairs population’, *American Journal of ophthalmology* 153(6), 1061–1066. 10.1016/j.ajo.2011.11.030 22330309

[CIT0035] Rensburg, R., 2021, ‘Healthcare in South Africa: How inequity is contributing to inefficiency’, *The Conversation*, p. 6, viewed 07 July 2021, from https://www.wits.ac.za/news/latest-news/opinion/2021.

[CIT0036] Schaumberg, D.A., Sullivan, D.A., Buring, J.E. & Dana, M.R., 2003, ‘Prevalence of dry eye syndrome among US women’, *American Journal of Ophthalmology* 136(2), 318–326. 10.1016/S0002-9394(03)00218-6 12888056

[CIT0037] Shah, S. & Jani, H., 2015, ‘Prevalence and associated factors of dry eye: Our experience in patients above 40 years of age at a Tertiary Care Center’, *Oman Journal of Ophthalmology* 8(3), 151–156. 10.4103/0974-620X.169910 26903719 PMC4738658

[CIT0038] Shanti, Y., Shehada, R., Bakkar, M.M. & Qaddumi, J., 2020, ‘Prevalence and associated risk factors of dry eye disease in 16 northern West bank towns in Palestine: A cross-sectional study’, *BMC Ophthalmology* 20, 1–8. 10.1186/s12886-019-1290-z 31931756 PMC6958733

[CIT0039] Sharma, A. & Hindman, H.B., 2014, ‘Aging: A predisposition to dry eyes’, *Journal of Ophthalmology* 2014(1), 781683. 10.1155/2014/781683 25197560 PMC4150485

[CIT0040] Shimazaki, J., 2018, ‘Definition and diagnostic criteria of dry eye disease: Historical overview and future directions’, *Investigative Ophthalmology & Visual Science* 59(14), DES7–DES12. 10.1167/iovs.17-23475 30481800

[CIT0041] Sidahmed, S., Geyer, S. & Beller, J., 2023, ‘Socioeconomic inequalities in diabetes prevalence: The case of South Africa between 2003 and 2016’, *BMC Public Health* 23(1), 324. 10.1186/s12889-023-15186-w 36788553 PMC9926686

[CIT0042] Stapleton, F., Alves, M., Bunya, V.Y., Jalbert, I., Lekhanont, K., Malet, F. et al., 2017, ‘Tfos dews ii epidemiology report’, *The Ocular Surface* 15(3), 334–365. 10.1016/j.jtos.2017.05.003 28736337

[CIT0043] Standl, E., Khunti, K., Hansen, T.B. & Schnell, O., 2019, ‘The global epidemics of diabetes in the 21st century: Current situation and perspectives’, *European Journal of Preventive Cardiology* 26(2_suppl), 7–14. 10.1177/2047487319881021 31766915

[CIT0044] Uchino, M. & Schaumberg, D.A., 2013, ‘Dry eye disease: Impact on quality of life and vision’, *Current ophthalmology reports* 1, 51–57. 23710423 10.1007/s40135-013-0009-1 PMC3660735

[CIT0045] Versura, P., Giannaccare, G. & Campos, E.C., 2015, ‘Sex-steroid imbalance in females and dry eye’, *Current Eye Research* 40(2), 162–175. 10.3109/02713683.2014.966847 25290221

[CIT0046] Wang, M.T., Muntz, A., Mamidi, B., Wolffsohn, J.S. & Craig, J.P., 2021, ‘Modifiable lifestyle risk factors for dry eye disease’, *Contact Lens and Anterior Eye* 44(6), 101409. 10.1016/j.clae.2021.01.004 33485806

[CIT0047] Wolffsohn, J.S., Arita, R., Chalmers, R., Djalilian, A., Dogru, M., Dumbleton, K. et al., 2017, ‘TFOS DEWS II diagnostic methodology report’, *The Ocular Surface* 15(3), 539–574. 10.1016/j.jtos.2017.05.001 28736342

[CIT0048] Yang, W., Luo, Y., Wu, S., Niu, X., Yan, Y., Qiao, C. et al., 2021, ‘Estimated annual economic burden of dry eye disease based on a multi-center analysis in China: A retrospective study’, *Frontiers in Medicine* 8, 771352. 10.3389/fmed.2021.771352 34926513 PMC8673084

[CIT0049] Yazdani, M., 2023, ‘Tear film lipid layer and corneal oxygenation: A new function?’, *Eye* 37(17), 3534–3541. 10.1038/s41433-023-02557-1 37138094 PMC10686381

[CIT0050] Yilmaz, U., Gokler, M.E. & Unsal, A., 2015, ‘Dry eye disease and depression-anxiety-stress: A hospital-based case control study in Turkey’, *Pakistan Journal of Medical Sciences* 31(3), 626. 10.12669/pjms.313.7091 26150857 PMC4485284

[CIT0051] Yu, J., Asche, C.V. & Fairchild, C.J., 2011, ‘The economic burden of dry eye disease in the United States: A decision tree analysis’, *Cornea* 30(4), 379–387. 10.1097/ICO.0b013e3181f7f363 21045640

[CIT0052] Zhang, X., Zhao, L., Deng, S., Sun, X. & Wang, N., 2016, ‘Dry eye syndrome in patients with diabetes mellitus: Prevalence, aetiology, and clinical characteristics’, *Journal of Ophthalmology* 2016(1), 8201053. 10.1155/2016/8201053 27213053 PMC4861815

[CIT0053] Zhou, Y., Murrough, J., Yu, Y., Roy, N., Sayegh, R., Asbell, P. et al., 2022, ‘Association between depression and severity of dry eye symptoms, signs, and inflammatory markers in the DREAM study’, *JAMA Ophthalmology* 140(4), 392–399. 10.1001/jamaophthalmol.2022.0140 35266971 PMC8914873

